# Prehospital transfusion strategies in haemorrhagic shock

**DOI:** 10.1186/s13049-026-01547-y

**Published:** 2026-01-22

**Authors:** Brian Burns, Brodie Nolan, Ian Ferguson, Michael Peddle, Christopher Partyka, Geoff Healy, Stacy Shackelford, Bryan Cotton

**Affiliations:** 1 Aeromedical Operations, NSW Ambulance, Sydney, Australia; 2 Faculty of Medicine and Health, Sydney University, Sydney, Australia; 3 Redline Resuscitation Institute, Sydney, Australia; 4 Emergency Department, Royal North Shore Hospital, Sydney, Australia; 5 Trauma Service, Royal North Shore Hospital, Sydney, Australia; 6 Faculty of Medicine and Health, University of NSW, Sydney, Australia; 7 Emergency Department, Liverpool Hospital, Sydney, Australia; 8 Department of Medicine, University of Toronto, Toronto, Canada; 9 Division of Emergency Medicine, Schulich School of Medicine and Dentistry, Western University, London, Canada; 10 Ornge, Mississauga, Canada; 11 Toronto, First60 Canada; 12 Emergency Department, St.Michael’s Hospital, Toronto, Canada; 13 Defense Health Agency, Falls Church, CO USA; 14 McGovern Medical School, UT Health Houston, Houston, TX USA; 15 Red Duke Trauma Institute, Memorial Hermann Hospital, Houston, TX USA

**Keywords:** Prehospital, Trauma, Bleeding, Transfusion, Haemostatic, MTP, Resuscitation, Whole blood

## Abstract

**Background:**

Massive transfusion protocols are established in-hospital practices for managing haemorrhagic shock, yet critical bleeding accounts for up to 40% of trauma deaths, with half occurring before hospital arrival. This has driven interest in prehospital transfusion, a concept originating in military settings where early blood product administration during prolonged evacuations demonstrated significant survival benefits. Despite compelling military evidence showing improved 24-h and 30-day survival with prehospital transfusion, civilian adoption faces substantial challenges. Global interest in this domain is increasing, particularly in regions with trauma populations remote from immediate access to major trauma centres.

**Main body:**

Current prehospital transfusion strategies have evolved toward low titre group O whole blood utilisation, which offers simplified logistics, reduced donor exposures, and physiologic component ratios. There is a gap in evidence in prehospital transfusion strategies with a paucity of RCTs comparing whole blood to component therapy. A recent Cochrane review included 18 RCTs and 5041 patients. For prehospital transfusion strategies 5 studies compared use of plasma (fresh frozen plasma (FFP) or lyophilised plasma) versus 'standard of care’, with uncertain effect of plasma on all-cause mortality at 24 h. Observational studies demonstrate four-fold increased survival rates with whole blood compared to component therapy and high-quality randomised controlled trials are ongoing. Implementation models vary from direct stocking at EMS stations to intercept and hospital-based approaches. Future research priorities include developing prediction scores for massive transfusion requirements, validating optimal physiological thresholds for transfusion initiation, and refining patient selection criteria. Comparative effectiveness research comparing whole blood against component therapy and alternative products is essential, as is evaluating injury pattern-based protocols. Technological innovations promise to address current limitations through artificial intelligence applications for predictive algorithms, universal blood products eliminating compatibility concerns, advanced storage technologies extending shelf life, drone-based delivery systems for remote access, and blockchain technology for enhanced traceability and safety.

**Conclusion:**

Prehospital transfusion represents a transformative paradigm shift in trauma care with potential to significantly reduce mortality worldwide. Success requires coordinated multi-stakeholder collaboration among EMS agencies, hospitals, blood suppliers, regulatory bodies, and research institutions. Continued focus on evidence-based practice, patient safety, and technological innovation will be essential to ensure life-saving blood products reach critically injured patients at the earliest possible moment, regardless of location.

## Background

Massive transfusion protocol (MTP) is an established in-hospital practice for the initial treatment and resuscitation of patients in hemorrhagic shock. Versions of this include the use of balanced component therapy, whole blood and viscoelastic testing (VET) guided transfusion. The aim of an MTP is to provide haemostatic resuscitation and restore blood circulating volume and improve tissue perfusion and oxygen delivery, whilst concurrent steps are taken to achieve haemorrhage control. It is estimated that critical bleeding accounts for up to 40% of trauma deaths, with a significant number of these being preventable with optimal treatment [1–3]. Moreover, death due to bleeding occurs early after injury with around half of trauma deaths related to bleeding occurring before they reach the hospital. Thus, it seems intuitive to commence blood transfusion as close to the point of injury as possible in order to sustain life.

The concept of prehospital transfusion is not new; it originated in military settings where the challenges of prolonged evacuation times and severe injuries necessitated early blood product administration. In a retrospective cohort study of 502 US military combat casualties undergoing medical evacuation, prehospital transfusion within 36 min after injury was associated with improved survival at 24 h (adjusted hazard ratio for mortality 0.26) and at 30 days (adjusted hazard ratio for mortality 0.39), while more delayed transfusion did not impact survival [4]. Compelling military evidence such as this has driven civilian adoption, though significant challenges remain. The use of prehospital blood transfusions as a tool for managing hemorrhagic shock has barriers to overcome before it becomes widely available to patients. In the US, recent estimates indicate that only about 1% of all EMS units located in 24 US states have implemented prehospital blood transfusion as of 2024 [5]. One of the potential reasons for this is proximity to a trauma centre and short transport times for many of these urban units. The growth in WB (or any product) in the prehospital setting has been in the longer transport areas (> 15 min), although EMS units in suburban areas have been increasingly implementing prehospital blood, albeit with no reimbursement/funding to do so. However, there is increasing global interest in performing high quality research in this domain [6, 7]. Many regions and jurisdictions, in both developing and developed countries, face the challenge of trauma patient populations in rural settings remote to major trauma centres and definitive haemorrhage control. These challenges often drive innovation. In this paper, we describe current prehospital transfusion techniques and strategies, key areas of future research in prehospital transfusion and potential future innovations in this domain.

## Main text

### Current prehospital transfusion strategies

As this was not a systematic review, rather a narrative review paper, our search strategy was flexible and iterative, without a rigid protocol. Our strategy was exploratory and focused on identifying key relevant literature in the topic without a comprehensive or exhaustive search. Key literature was identified in two ways- MeSH terms in PubMed targeting prehospital transfusion strategies published in the last 10 years. Co-authors suggestions of references were also considered by the lead author (BB) during the iterative writing of the manuscript. Two of these authors have an extensive academic track record in blood transfusion in trauma.

There is a gap in evidence in prehospital transfusion strategies with a paucity of RCTs comparing whole blood to component therapy. A recent Cochrane review of blood transfusion strategies for major bleeding in trauma included 18 RCTs and 5041 patients. For prehospital specific transfusion strategies 5 studies compared use of plasma (fresh frozen plasma (FFP) or lyophilised plasma) versus 'standard of care’, with uncertain effect of plasma on all-cause mortality at 24 h [8]. The evolution of prehospital transfusion has been marked by a shift toward WB utilisation. Use of low titre group O whole blood (LTOWB) as the first-choice blood product for treatment of patients with traumatic life-threatening bleeding in the prehospital phase of resuscitation is increasing but not supported yet by high quality trials. LTOWB offers several potential advantages over component therapy in the prehospital setting. One trial of nearly 1,400 trauma patients receiving emergency blood products prehospital or in the emergency department found that those who received WB had a four-fold increased survival rate and were also less likely to need more blood later [9]. The finding that WB patients had similar crude survival (75% vs 76%) but fourfold adjusted survival odds is paradoxical and suggests the statistical model may be over-correcting or that unmeasured confounders favoured the WB group. Prehospital whole blood programmes have demonstrated feasibility and improved physiology [10]. However, these studies are limited by their retrospective, observational nature, and as such the treatment effects and direction need to be interpreted with caution. Nonetheless, the practical advantages of WB in prehospital settings include simplified logistics, reduced complexity of administration, reduced number of donor exposures and the provision of all blood components in physiologic ratios. In addition, a recent multi-centre in-hospital study noted that early use of LTOWB increased the likelihood of achieving balanced ratios during the first four hours of resuscitation, which was associated with increased survival [11]. This study should be interpreted with caution given it’s observational nature, lack of randomisation and risk of selection bias and confounders. That accepted, it seems likely that the prehospital setting is where the marginal benefits of LTOWB are greatest. Prehospital LTOWB is however not standard care in most jurisdictions as yet and is the subject of two current RCTs, one of which (TOWAR) recently completed recruitment [12, 13].

While there is evidence favouring LTOWB use in trauma, the evidence is lacking in other areas such as major obstetrical or gastrointestinal bleeding and logistical issues currently limit its widespread deployment in some prehospital environments. However, in each of these major haemorrhage scenarios, patients are all bleeding the same thing, whole blood. Given controversy and logistical changes, many prehospital services are still reliant on packed red blood cells (PRBC) with or without extended life plasma (ELP) or other blood component options to address major haemorrhage in the prehospital setting [14]. These include options such as prothrombin complex concentrate (PCC), fibrinogen concentrate (FC) and freeze dried plasma (FDP) which are available in many prehospital settings. As a result, current prehospital transfusion protocols vary significantly across EMS systems, while each generally focusing on physiological indicators of hemorrhagic shock as triggers for intervention. Regardless of available products, a key central tenet to every protocol should be early recognition of the need for transfusion and once identified, rapid intervention.

Various implementation models are used for the provision of prehospital blood products. The direct stocking model establishes blood product storage at EMS stations, allowing for immediate availability but requiring sophisticated cold chain management and tight governance. The intercept model entails emergency services (e.g. police) carrying blood products from a hospital blood bank to rendezvous with the EMS agency on scene or en route to hospital. The hospital-based model entails provision of blood products to EMS personnel from the hospital blood bank on a case-by-case basis. Generally, more mature systems utilise the direct stocking model. A combined model developed in New South Wales, Australia, includes direct stocking of HEMS bases augmented by an additional retrieval transfusion protocol (RTP) which allows the EMS coordinating centre to activate the dispatch and delivery of further blood products from a local hospital blood bank to the scene or to the prehospital medical team en route to a trauma centre with an exsanguinating patient; essentially a prehospital Massive Transfusion Protocol (MTP). However, due to the urgent nature of this protocol, and need to source products from a network of local blood banks, most of which do not stock extended life (thawed or liquid) plasma or platelets, this generally results in transfusion strategies dominated by the use of PRBCs. This leads to higher likelihood of early unbalanced ratios of plasma and platelets, which are associated with significantly higher mortality rates [11]. Despite this, RTP gives patients who otherwise would have exsanguinated a bridge to survive until definitive haemorrhage control, in particular with long prehospital distances [15]. This raises the question of ethical frameworks, cost-effectiveness and health policy considerations of prehospital transfusion strategies. Ethical frameworks are centred around equity of timely access to blood products to as many critically bleeding patients as possible, in particular for patients in rural/regional locations, remote from trauma centres and definitive haemorrhage control. In each jurisdiction this should be viewed within the sphere of government health policy and systems and a cost-effectiveness health economics analysis. Cost effectiveness analyses should include Incremental Cost-Effectiveness Ratio(ICER), Willingness To Pay (WTP) thresholds and Quality-Adjusted Life Years (QALYs). There is a big gap in research in this area. A key factor to consider for implementation of prehospital transfusion strategies that also affects cost-effectiveness are the regulatory, legal, and funding challenges, particularly for civilian EMS systems. For example, in the US, one of the reasons many EMS units do not carry blood products is that they receive no funding or reimbursement for carrying and is hence a negative cost, despite potential patient benefit. In many European countries, the entire EMS system, including HEMS is publicly funded as part of the healthcare system with blood products supplied through the national blood service, as it is in the hospitals. The challenge in Europe and Australia is less about reimbursement and more about managing supply chain costs and wastage. Supply cold chain integrity is a significant challenge in prehospital transfusion. In Canada, regulatory constraints have meant that prehospital transfusion is a relatively recent development where only 11 of 30 critical care transport bases have a blood transfusion programme [16]. This adoption has been limited due to scope of practice restrictions of most land-based paramedics, more logistically favourable products, e.g. lyophilised plasma is not approved for use in Canada (or Australia) and there’s also a lack of standardised national guidelines [17].

### Future research priorities in prehospital transfusion

Future research should incorporate the development and validation of prehospital massive transfusion prediction scores and identification of optimal physiologic thresholds for transfusion initiation. We should also seek to evaluate injury pattern-based protocols. Rigorous assessment of the role of point-of-care diagnostic tools to determine transfusion trigger and product type (e.g. clotting factor or clot performance deficiencies) needs to be performed. Bleeding is a time sensitive pathophysiological process and thus identification of the effective time to transfusion and planning timeline should be studied. Lastly, discovery of storage methods to extend blood product shelf life and the development of blood substitutes is a key priority in prehospital transfusion science with respect to austere environment, equity of access and low/middle income countries.

#### Optimisation of patient selection criteria

One of the most critical research priorities involves refining criteria to identify patients who will benefit most from prehospital transfusion. A key research question is what are the benefits and harms of different strategies (therapeutic, logistical, or both combined) and interventions (WB, PRBC, ELP, platelet, FC, PCC, FDP etc.) for patients in prehospital hemorrhagic shock [18]. In addition, prehospital clinicians ideally require rapid and accurate methods for the identification of conditions that mimic haemorrhagic shock (including brain injury associated shock, [19] tension pneumothorax, etc.) to avoid transfusion in trauma patients in whom it may prove unnecessary and deplete a limited resource. However, there is evolving evidence that demonstrates improved survival in those with brain injury receiving plasma and WB in the early resuscitative phase, suggesting that even those severely injured patients without hemorrhagic shock may benefit [20, 21].

#### Blood product selection

On overview of prehospital blood products is presented in Table 1. As previously noted, access to LTOWB is not universal nor will it be feasible for all programs based on patient populations serviced along with the associated operational and logistical issues. Recognising the wastage challenges with thawed plasma, alternative products such as FDP, PCC and FC which have prolonged shelf life and less stringent storage requirements, may be able to fill the gap for patients requiring MTP. However, the reconstitution of such products can represent a logistical challenge for pre-hospital teams with limited personnel. A focus comparing different treatment algorithms will allow programs to adapt the most appropriate solution for their regional and operational realities. Development of longer shelf-life and room-temperature stable products is essential. Many US centres utilise never-frozen liquid plasma to address wastage with five-day shelf-life of thawed plasma. This product extends the shelf-life of plasma to 21–26 days with factor levels and activity comparable to that of immediately thawed plasma [22]. A focus comparing different treatment algorithms will allow programs to adapt the most appropriate solution for their regional and operational realities.

**Table 1:** Potentially Utilised Prehospital Blood Products

Strategy	Composition	Storage Requirements	Advantages for PH Use	Limitations for PH Use
LTOWB	Complete blood with RBCs, plasma, platelets, and coagulation factors in physiologic ratios	2–6 °C; 35-day shelf life (cold-stored); Requires continuous temperature monitoring	Single-unit simplicity reduces cognitive load. Balanced resuscitation in one product. Platelets present (though reduced function when cold-stored). Universal donor compatibility. Reduced volume/transfusion burden. Simplified logistics vs. multiple components. Lower risk of dilutional coagulopathy	Limited availability in many jurisdictions. Not approved in all countries. Cold storage platelet dysfunction. Requires significant refrigeration infrastructure. Higher wastage costs if unused. Blood typing compatibility concerns in massive transfusion. Short shelf life limits stockpiling
PRBC	Concentrated RBCs with ~ 70% of plasma removed; Hct 55–60%	2–6 °C; 35–42 day shelf life; Requires continuous temperature monitoring	Universally available through blood banks.Well-established safety profile. Improves oxygen-carrying capacity. Can use O-negative for universal compatibility. Established regulatory framework. Rotation programs with hospitals possible	Provides no coagulation factors or platelets. Must be combined with other products for balanced resuscitation. Increased complexity managing multiple products. Contributes to dilutional coagulopathy if used alone. Requires refrigeration. Higher wastage rates when carrying multiple products
Extended Life Plasma (ELP)	All coagulation factors, proteins, and immunoglobulins in physiologic concentrations; previously frozen plasma that has been thawed	2–6 °C refrigerated storage; 5-day shelf life once thawed; Ready to administer (no thaw time required)	Contains all coagulation factors. No thaw time—immediately available. Addresses coagulopathy. Well-established evidence base. Established safety profile. Can be pre-thawed and stocked in refrigerator. ABO compatible plasma readily accessible. Volume expander (200–250 mL/unit)	Very short shelf life once thawed (5 days). Requires refrigeration infrastructure. High wastage rates in low-volume prehospital services. ABO compatibility required for optimal use. Requires coordination with blood bank for rotation.Heavy and bulky to transport. Factor V and VIII levels decline over 5-day period
Freeze Dried Plasma (FDP)	Lyophilized plasma (FLyP, LyoPlas) containing all coagulation factors; reconstituted with sterile water	Room temperature storage (up to 37 °C); 2-year shelf life; Reconstitution: 3–6 min with sterile water	No refrigeration required. Extended 2-year shelf life. Lightweight and compact. Rapid reconstitution (3–6 min). Reduced wastage. Well-suited for austere/remote environments. Military proven (French FLyP, German LyoPlas). Contains all coagulation factors	Not approved in all jurisdictions (e.g., Canada, USA). Limited civilian availability. Requires sterile water and mixing time. Potential for reconstitution errors. Limited evidence in civilian prehospital setting. More expensive than traditional plasma. Requires specific training for reconstitution. ABO compatibility considerations remain
Prothrombin Complex Concentrate (PCC)	Concentrated vitamin K-dependent factors (II, VII, IX, X); also contains proteins C and S	Room temperature or refrigerated (depending on formulation); 2–3 year shelf life; Reconstitution required (typically < 5 min)	Small volume (typically 20–50 mL). No refrigeration needed. Rapid reconstitution. Extended shelf life. Rapid correction of coagulopathy. Minimal wastage. Low storage burden. Used successfully in some Canadian HEMS programs (STARS)	Does not replace fibrinogen or platelets. Limited factors (only II, VII, IX, X). Thrombotic risk concern. Off-label use for trauma (approved for warfarin reversal). Higher cost per dose. Requires adjunct therapy (fibrinogen, platelets). Limited prehospital evidence base. Dose uncertainty in trauma. Cannot provide volume resuscitation
Fibrinogen Concentrate (FC)	Purified human fibrinogen (typically 1 g powder); Two formulations: Riastap and Fibryga	Riastap: Refrigerated storage 2–8 °C (do not freeze); Protect from light; 2–3 year shelf life. Once reconstituted: store below 25 °C and use within 6 h Fibryga: Storage at 2–25 °C (room temperature acceptable); Do not freeze; Protect from light; 36 months shelf life. Once reconstituted: stable for 24 h at 25 °C. Both require reconstitution (~ 5–10 min)	Targeted treatment of fibrinogen deficiency. Small volume (50 mL reconstituted). Fibryga**:** No refrigeration required—room temperature storage. Fibryga: Longer reconstituted stability (24 h vs 6 h). Extended shelf life (up to 36 months). Rapid fibrinogen repletion. Low storage footprint. Minimal wastage. Point-of-care viscoelastic testing can guide use Riastap: Once reconstituted can be stored at room temperature (< 25 °C) for 6 h	Treats only ONE component of coagulopathy. Does NOT replace other factors or platelets. High cost. Requires viscoelastic monitoring for optimal use. Limited prehospital evidence. Off-label use in most jurisdictions. Cannot provide volume expansion. Reconstitution adds delay. Requires comprehensive coagulation management strategy. Riastap: Requires refrigeration for unreconstituted storage Riastap: Shorter reconstituted stability (6 h)
Cold Stored Platelets(CSPs)	Apheresis or pooled buffy-coat derived platelets; stored in platelet additive solution (PAS); pathogen-reduced when available	1–6 °C refrigerated; 14-day shelf life (FDA approved); No agitation required	Superior haemostatic function vs. room temperature platelets Lower bacterial contamination risk. No agitation or bacterial testing required.Can be stored with RBCs/plasma in same cold chain). Extended shelf life (14 days vs. 5 days). Approval only for active bleeding when conventional platelets unavailable/impractical. Military proven	Shorter circulation time in vivo Limited civilian availability Limited prehospital evidence (trials ongoing—CriSP-HS, CriSP-TBI) Not approved in all jurisdictions

#### Comparative effectiveness research

Comparative benefits and harms of different protocols represent a fundamental research gap that require large-scale high quality prospective trials. Many time-critical interventional trials suffer from being underpowered due to unexpected effects in the intervention and standard arms or by not meeting the target number and often contain a heterogenous patient population or the intervention is occurring too late, or the analysis does not account for the variable time to intervention, as is often the case in trauma. Perhaps, it is time to move away from single intervention studies in trauma related bleeding towards adaptive platform trials. These trial designs should also contain whole-system cost-effectiveness analysis and consumer input from the outset. Part of these adaptive trial designs could be to examine certain patient populations (paediatric, geriatric) and locations (urban versus rural/remote). Additionally, analyses comparing prehospital intervention to in-hospital intervention without incorporating the effect of time to intervention often generates inaccurate conclusions.

#### Implementation science research

Key implementation research priorities include training and education, economic evaluation and system integration. The limited studies performed demonstrate blood transfusion training for prehospital providers is feasible and associated with short-term improvements in knowledge, skills, and confidence [23]. However, there is inconsistent instructional design and skill sustainment and limited evaluation of long-term clinical outcomes.

#### Technology integration research

Emergency technologies should be tested under a research protocol. These include point-of-care coagulation and viscoelastic testing devices, AI for decision support and transfusion trigger accuracy and real-time telemedicine integration between the prehospital and hospital system. Outcome measurement and data collection. A lack of centralised international database for prehospital haemorrhagic shock and in-hospital patient outcomes represents a significant barrier to research advancement. Priorities in this area includes: development of standardised outcomes measures, databases matching prehospital and in-hospital care, international networks and long term follow up methodologies.

### Future innovations in prehospital transfusion

#### Technological

Emerging diagnostic technologies promise to revolutionise prehospital transfusion by enabling rapid assessment and personalised treatment decisions. Advanced point-of-care devices incorporating microfluidics, electrochemical sensing, and fluorescent microscopy are being developed for prehospital use. Focused point of care echocardiography (transthoracic and transoesophageal) is also likely to increase as a clinical tool to assess the cardiovascular system during severe haemorrhage. These innovations could enable rapid blood type determination, real-time coagulation assessment, haemoglobin monitoring and prediction of transfusion requirements. The practical implications of such testing would need to be evaluated carefully to ensure that it contributes not just to knowledge of the patient’s physiology, but also to an improvement in meaningful outcomes.Virtual reality(VR) based training systems could provide standardised, repeatable training experiences for EMS personnel to simulate transfusion scenarios, orders of priority, equipment familiarisation, assessment and certification tools [24]. Augmented reality (AR) technology could provide real-time guidance during actual transfusions: step-by-step procedure guidance for non-experts, patient identification verification, equipment setup assistance and real-time consultation with medical oversight.

#### Adjunctive vasopressor therapy

The sympathoinhibitory phase of hemorrhagic shock is characterised by late arterial hypotension with bradycardia and vasodilation that is refractory to endogenous catecholamines [25]. It is poorly understood in humans but supported in animal studies where simple replacement of intravascular volume fails to restore diastolic filling pressures in severe haemorrhage [26]. Exogenous vasoactive therapy may be of benefit here. As a result of the evolving literature, renewed interest in the early use of vasopressor agents to support hemodynamics has appeared [27]. In particular, vasopressin has been identified as a potential answer to vasodilatory states and distributive shock after injury and exaggerated inflammatory responses. Vasopressin (likely through splanchnic vasoconstriction similar to that observed in gastrointestinal bleeding) has been associated with improved survival in uncontrolled abdominal haemorrhage [28].

However, existing data from randomised trials on the effects of vasopressor therapy following traumatic injury is limited [29], or of very low quality [30]. As a result, there is a strong justification to undertake clinical trials on early vasopressor use during trauma resuscitation examining the role on the optimisation of perfusion, restriction of blood product administration and overall mortality. Based off of preliminary data from Sims et al. who found reduced transfusion requirements in hemorrhagic shock patients receiving vasopressin early in their resuscitation [29], the LITES network is currently performing a multicentre, RCT (CAVALIER) of early vasopressin administration in patients presenting with evidence of haemorrhage [31].

#### Artificial Intelligence(AI) and Machine Learning(ML)

AI and ML are being used to predict blood supply and demand, helping blood banks to plan collections and manage their resources effectively. In prehospital settings, AI applications could include predictive algorithms for massive transfusion requirements, optimal blood product selection algorithms, resource allocation optimisation and real-time decision support systems.

#### Universal blood products

Research into universal blood products could eliminate compatibility concerns and simplify prehospital protocols. These approaches hold the potential to mitigate blood shortages, enhance transfusion safety, and transform patient care by removing the constraints of blood type compatibility. Three primary approaches show promise: Enzymatically treated red blood cells to remove ABO antigens [32], red blood cells derived from induced pluripotent stem cells [33] and artificial oxygen carriers as blood substitutes [34].

#### Advanced blood storage technologies

Innovations in blood storage and preservation could extend shelf life and improve product availability: these include novel preservation solutions, advanced cold chain technologies, freeze-dried blood products and lyophilized plasma preparations. Advanced storage systems incorporating Internet of Things (IoT) technology could optimize blood product management by performing real-time temperature monitoring, automated inventory management, predictive maintenance systems and integration with hospital information systems.

#### Delivery system innovations

Drone-based blood delivery (DBD) which involves the creation of a service network, with a a blood bank storage hub and drones as the delivery vehicles, addresses blood delivery challenges in remote or difficult-to-access locations [35]. Key advantages of drone delivery include rapid response to remote locations, reduced transport time, access to areas with challenging terrain and cost-effectiveness for specific scenarios.

#### Communication and coordination technologies

Real-Time Communication Systems: enhanced communication technologies could improve coordination between EMS, blood banks, and receiving hospitals with real-time blood inventory sharing, automated hospital notification system, telemedicine consultation platforms and mobile communication apps for EMS personnel.

Blockchain Technology: blockchain applications could enhance blood product traceability and safety by ensuring immutable transfusion records, supply chain verification, quality assurance tracking and patient consent management.

## Conclusions

Prehospital transfusion represents a transformative approach to trauma care with the potential to save thousands of lives annually. Current evidence, particularly from military settings, demonstrates clear survival benefits, and civilian programs and research are showing promising results. However, significant challenges remain in implementation, including regulatory barriers, economic constraints, and logistical complexities. Future research must focus on optimising patient selection criteria, comparing different transfusion strategies, and developing implementation frameworks that can be adapted across diverse EMS systems. Technological innovations offer exciting possibilities for improving safety, efficiency, and outcomes of prehospital transfusion. From artificial intelligence applications to novel blood products and automated systems, these innovations could address many current limitations and expand the accessibility of life-saving transfusion therapy.

The success of prehospital transfusion programs will ultimately depend on coordinated efforts among multiple stakeholders, including EMS agencies, hospitals, blood suppliers, regulatory bodies, and research institutions. The Prehospital Blood Transfusion Initiative Coalition in the US and the Traumatic Hemorrhage Outcomes Research (THOR) Network represent an important model for such collaboration.

As the field continues to evolve, maintaining a focus on evidence-based practice, patient safety, and continuous quality improvement will be essential. The goal is simple: to ensure that life-saving blood products are available to critically injured patients at the earliest possible moment, regardless of their location or the circumstances of their injury.

The paradigm shift toward prehospital transfusion represents not just a change in technique or protocol, but a fundamental reimagining of trauma care that extends the hospital's capabilities into the field. With continued research, innovation, and implementation efforts, prehospital transfusion has the potential to significantly reduce trauma mortality and improve outcomes for injured patients worldwide.

## Data Availability

N/A.

## Abbreviations

AI: Artificial intelligence
AR: Augmented reality
DBD: Drone based delivery
ELP: Extended life plasma
EMS: Emergency medical service
FC: Fibrinogen concentrate
FDP: Freeze dried plasma
IoT: Internet of things
LTOWB: Low titre group O whole blood
ML: Machine learning
MTP: Massive transfusion protocol
PCC: Prothrombin complex concentrate
PRBC: Packed red blood cells
RTP: Retrieval transfusion protocol
VET: Viscoelastic testing
VR: Virtual reality
